# *Bifidobacterium breve* reduces apoptotic epithelial cell shedding in an exopolysaccharide and MyD88-dependent manner

**DOI:** 10.1098/rsob.160155

**Published:** 2017-01-25

**Authors:** K. R. Hughes, L. C. Harnisch, C. Alcon-Giner, S. Mitra, C. J. Wright, J. Ketskemety, D. van Sinderen, A. J. M. Watson, L. J. Hall

**Affiliations:** 1 The Gut Health and Food Safety Programme, Institute of Food Research, Norwich Research Park, Colney, Norwich NR4 7UA, UK; 2 Norwich Medical School, University of East Anglia, Norwich Research Park, Norwich NR4 7UQ, UK; 3 APC Microbiome Institute, University College Cork, Cork, Ireland; 4 School of Microbiology, University College Cork, Cork, Ireland

**Keywords:** *Bifidobacterium*, epithelial cell shedding, inflammatory bowel disease, exopolysaccharide

## Abstract

Certain members of the microbiota genus *Bifidobacterium* are known to positively influence host well-being. Importantly, reduced bifidobacterial levels are associated with inflammatory bowel disease (IBD) patients, who also have impaired epithelial barrier function, including elevated rates of apoptotic extrusion of small intestinal epithelial cells (IECs) from villi—a process termed ‘cell shedding’. Using a mouse model of pathological cell shedding, we show that mice receiving *Bifidobacterium breve* UCC2003 exhibit significantly reduced rates of small IEC shedding. Bifidobacterial-induced protection appears to be mediated by a specific bifidobacterial surface exopolysaccharide and interactions with host MyD88 resulting in downregulation of intrinsic and extrinsic apoptotic responses to protect epithelial cells under highly inflammatory conditions. Our results reveal an important and previously undescribed role for *B. breve*, in positively modulating epithelial cell shedding outcomes via bacterial- and host-dependent factors, supporting the notion that manipulation of the microbiota affects intestinal disease outcomes.

## 1. Introduction

Bifidobacteria represent one of the first colonizers of the infant gut and are prominent members of the adult gut microbiota [1,2]. They have been linked to a number of health-promoting activities, including the promotion of anti-tumour immunity [3], modulation of antimicrobial activities against pathogenic bacteria [4] and protection against relapse of ulcerative colitis [5,6]. Despite these purported benefits, the molecular mechanisms underlying these protective effects by bifidobacteria remain largely unknown, although recently components of their surface, including the exopolysaccharide (EPS), have been shown to play a significant role in modulating protective effects [7]. It is critical to obtain detailed insights into the mode of action by which microbiota members sustain and improve host health, as this will be central to future disease treatment/prevention strategies.

There is a growing body of evidence suggesting that the microbiota influences intestinal epithelial cell (IEC) function, including gene expression, cell division and energy balance [8–11]. These symbiotic bacterial/host relationships have co-evolved to the extent that the microbiota is indispensable for the maintenance of gut homeostasis [12]. Importantly, microbial dysbiosis, as indicated by a reduction in overall diversity, including specific reductions in *Bifidobacterium*, has been linked to inflammatory bowel disease (IBD) [13–15], underlining the critical importance of host/microbe interactions in maintaining a steady state within the intestine.

The epithelium of the small intestine represents the first line of defence against entry of bacteria into host tissues. Cell division in the crypt, under physiological conditions, is counterbalanced by cell shedding from the villi to maintain homeostasis and integrity of the crypt/villus axis. When the epithelial cell is shed, a discontinuity in the villus epithelial monolayer is created, which potentially compromises the epithelial barrier. In health, epithelial barrier function is maintained [16], owing to a dramatic redistribution of apical junction complex proteins, including zonula occludin 1 (ZO-1), occludin 1 and E-cadherin, which form a funnel that surrounds the shedding cell and plugs the resulting gap until the movement of neighbouring epithelial cells restores epithelial continuity [17–19].

TNF-α is a key cytokine in IBD. We and others have shown that TNF-α induces apoptosis of villus tip epithelial cells causing excessive shedding, leading to breakdown of the epithelial barrier and microulceration [16,20]. Delayed repair of epithelial defects caused by excessive cell shedding contributes to the development of macroscopic ulceration [21]. Our studies with confocal endomicroscopy of patients with IBD in clinical remission have demonstrated that those patients with high rates of cell shedding are more likely to relapse than those with low shedding rates, demonstrating a causative link between barrier function and the inflammatory response [21].

Given reports of beneficial effects of certain members of the gut microbiota in IBD and potential roles of microbial dysbiosis in these diseases, we hypothesized that certain health-promoting microbiota members, including *Bifidobacterium*, may play a role in protecting against the cell shedding response by modulating IEC function. To determine the contribution of bifidobacteria in cell shedding, we employed a well characterized *in vivo* mouse model in which pathological cell shedding is induced by intraperitoneal (IP) administration of lipopolysaccharide (LPS), driving mononuclear cell expression of TNF-α and subsequent caspase-3-positive shedding cells [22]. Our results suggest a particular bifidobacterial strain (i.e. human isolate *B. breve* UCC2003) positively modulates the small intestinal cell shedding response via host MyD88- and bacterial EPS-dependent interactions which serve to significantly reduce apoptotic signalling in the epithelial compartment. These data identify a previously unknown mechanism by which *Bifidobacterium* protects its host against pathological cell shedding. These findings may thus have important implications for the future design of therapeutic strategies in the context of intestinal diseases.

## 2. Material and methods

### 2.1. Animals

C57 BL/6 Jax mice (6–10 weeks) were obtained from Charles River. Vil-cre MyD88 transgenic mice (i.e. Cre recombinase expression causes truncation and resulting non-function of the MyD88 protein in IECs) were obtained from the Wellcome Trust Sanger Institute (kind gift from S. Clare).

### 2.2. Bacterial culture and inoculations

*Bifidobacterium breve* strains UCC2003, UCC2003del and UCC2003inv were used for animal inoculations. These strains and corresponding culturing conditions have been previously described in detail [7]. In brief, colonies were established from frozen glycerol stocks onto reinforced clostridial agar (RCA) plates before being subcultured into reinforced clostridial medium and subsequently Man Rogosa Sharpe medium (Oxoid, Hampshire) under anaerobic conditions. Bacteria were then purified by centrifugation and washed in PBS containing l-cysteine before being reconstituted in sterile PBS at a final concentration of approximately 1 × 10^10^ bacteria ml^−1^. 0.1 ml of inoculum was then administered to mice by oral gavage in 3 × 24 h doses followed by plating of faecal pellets on RCA containing 50 mg l^−1^ mupirocin to confirm stable colonization. Control mice received oral gavage of PBS only.

### 2.3. Lipopolysaccharide injections and tissue collections

Twenty-four hours after the last doses of *B. breve* or PBS control, mice received an IP injection of 1.25 mg kg^−1^ LPS from *Escherichia coli* 0111:B4 (Sigma) or sterile saline (control) and mice were sacrificed 1.5 h post-challenge with LPS. Proximal small intestine was collected in 10% neutral buffered formalin saline (Sigma) and fixed for 24 h followed by paraffin embedding. Samples of proximal small intestine were also collected into RNA Later (Qiagen) for transcriptome analysis or frozen on dry ice for subsequent ELISA analysis. In some cases, proximal small intestine was also collected into Hanks buffered saline solution (HBSS) for isolation of IECs.

### 2.4. Immunohistochemistry

Sections (5 µm) of paraffin-embedded small intestinal tissue were sectioned and used for immunohistochemistry. Following de-parafinization and rehydration, tissue sections were treated with 1% hydrogen peroxide in methanol to block endogenous peroxidases. Subsequently, slides were treated using heat-induced antigen retrieval in 0.01 M citrate acid buffer (pH 6) followed by incubation with a rabbit polyclonal anti-active caspase-3 (CC3) antibody (AF835: R&D Systems). Visualization of caspase-3 positivity was via a peroxidase-labelled anti-rabbit EnVision secondary antibody (Dako) and 3,3′-diaminobenzidine followed by counterstaining with haematoxylin. For macrophage staining, an antibody against F4/80 antigen (ab6640: Abcam) was employed using biotinylated anti-rat (BA-9401) and avidin–biotin reagent (PK-6100; Vector Laboratories).

### 2.5. Quantification of caspase-3 positivity

IECs were counted on a cell positional basis from villus tip (cell position (CP) 1) down towards the crypts under 400× magnification. Twenty well-orientated hemi-villi were counted per mouse and analysed using the Score, WinCrypts [23] and PRISM analysis software. IECs were defined as ‘normal’ in cases where staining for active caspase-3 was absent. Immunolabelled cells with either unaltered or shedding morphology were treated as caspase-3 positive. Imaging was performed with an Olympus BX60 microscope and C10plus digital camera.

### 2.6. RNA isolation and real-time polymerase chain reaction

Samples fixed in RNAlater solution were processed through RNeasy plus mini spin columns to isolate total RNA (Qiagen). In brief, samples were homogenized using a rotor stator hand held homogenizer in buffer RLT before processing through a QIAshredder column and subsequently RNeasy mini-spin columns. Purified RNA was eluted into RNAase free water. Reverse transcription was performed using the Quantitect reverse transcription kit (Qiagen) and cDNA used for real-time (RT-)PCR analysis. For RT-PCR, transcripts were amplified using Quantifast SYBR green mastermix (Qiagen) and Quantitect primer assays for TNF-α, TNF-R1 and F4/80 (EMR1). Expression of the housekeeping gene hypoxanthine–guanine phosphoribosyltransferase (HPRT) 5′-GACCAGTCAACAGGGGACAT-3′ (sense) and 5′-AGGTTTCTACCAGTTCCAGC-3′ (antisense) [24] was also determined. Cycling was performed on a Roche LightCycler 480 using the following conditions: 95°C, 5 min then 40 cycles of 95°C, 10 s; 60°C, 35 s. Relative quantification of levels of transcript expression was calculated using the Pfaffl method [25] by comparing cycle threshold (CT) value of each target gene to the CT value of housekeeper. Data are presented as a ‘fold change’ in expression (normalized against control untreated mice per cells).

### 2.7. Isolation of intestinal epithelial cells and FACS analysis

IECs were isolated using a modification of the Weiser methodology [26]. In brief, whole small intestine was collected in ice-cold HBSS before being chopped into 0.5 cm^2^ pieces and washed in a solution containing 0.154 M NaCl and 1 mM DTT, and subsequently a solution containing 1.5 mM KCl, 96 mM NaCl, 27 mM tri-sodium citrate, 8 mM NaH_2_PO_4_ and 5.6 mM Na_2_HPO_4_, pH 7.3. IECs were then isolated by incubation in PBS containing 1.5 mM EDTA and 0.5 mM DTT, shaking at 200 r.p.m. and at 37°C. Purity of epithelial preparations was confirmed by histological analysis of stripped intestinal mucosa and by FACS analysis of isolated cells. For FACS analysis, 5 × 10^6^ cells were stained with anti-mouse CD45-A700 (Biolegend) on ice for 30 min. After two washes in HBSS containing 0.01 BSA, 2 mM EDTA, 20 mM HEPES and 0.01% NaN_3_, propidium iodide was added (Biolegend) and samples analysed on a Sony FCS SH-800 flow cytometer. Data were analysed using FlowJo (TreeStar).

### 2.8. ELISA

Frozen proximal small intestinal samples were homogenized in extraction buffer containing protease inhibitors (Roche), cleared by centrifugation and analysed using a commercial ELISA kit for TNF-α (eBioscience) as per manufacturer's protocol. Measurement of TNF-α immunoreactivity was at 450 nm, using a Fluostar Optima plate reader (BMG Labtech).

### 2.9. SDS–PAGE and Western blotting

Isolated IECs were lysed in CelLytic MT reagent (Sigma) before centrifugation at 10 000 rpm for 10 min to pellet cellular debris. Supernatants were mixed with 2 × Laemmli sample buffer before being separated by sodium dodecyl sulfate (SDS)–PAGE with 3–14% acrylamide gel and transferred to Hybond-P PVDF membrane (GE Healthcare, Buckinghamshire, UK) and blocking with 5% Marvel milk in with tris(hydroxymethyl)aminomethane (Tris). (Tris)-buffered saline containing Tween 20 (TTBS) immunostaining was performed with 1/1000 anti-TNF-R1 antibody (Abcam) and 1/5000 goat anti-Rabbit IgG HRP conjugate (Millipore) on a reduced gel. Macrophage expression was analysed similarly using antibody against F4/80 antigen (Abcam) at 1 : 1000 and goat anti-rat IgG-HRP (SantaCruz, at 1 : 3000), on a non-reduced gel. Washes were in TTBS. For detection, Immobilon Western chemiluminescent HRP substrate (Millipore) was applied to the membrane as recommended by the manufacturer and signal was detected, using a FluorChem E imaging system (Protein Simple). Band densities were quantified using Fiji [27].

### 2.10. Polymerase chain reaction array analysis

Real-rime ready Custom Panel 480–96+ PCR arrays were obtained (Roche) and quantitative PCR analysis performed. RNA was extracted from whole small intestinal tissue preserved in RNAlater reagent (Sigma), using RNeasy plus mini kits (Qiagen). Reverse transcription was performed, using Transcriptor First Strand cDNA Synthesis Kit followed by analysis of targets using LightCycler 480 Probes Master on a LightCycler 480 platform (all Roche). Standard protocols as per manufacturer recommendations were followed. CT values of target genes were normalized to expression of the housekeeping gene HPRT and fold change versus control samples calculated using the delta/delta CT method [25].

### 2.11. Statistical analysis

Experimental results were plotted and analysed for statistical significance with Prism v. 5 software (GraphPad Software). A *p*-value of less than 0.05 was used as significant in all cases.

## 3. Results

### 3.1. Lipopolysaccharide induces cell shedding from small intestinal villi in a dose-dependent manner

**Figure 1.**
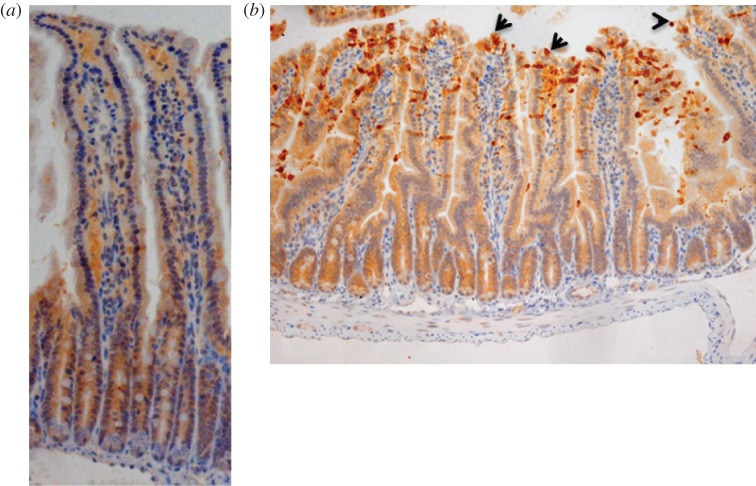
LPS challenge induces cell shedding from small intestinal villi. C57 BL/6 mice were administered either (*a*) PBS (control) or (*b*) LPS by IP injection and proximal small intestines removed after 1.5 h. Processed tissue was sectioned and stained by immunohistochemistry for CC3 (i.e. brown cells indicate shedding event), also highlighted by arrows. A representative picture for each group is shown (12 mice per group, two independent experiments).

Caspase-3 is activated in IECs during their extrusion from the tips of small intestinal villi [18,28]. Similar to previous reports, we found that control C57 BL/6 mice receiving IP PBS injection showed low levels of cell shedding as evidenced by low level expression of cleaved caspase-3 (CC3) in the epithelial cell layer (figure 1*a*). Recent studies have demonstrated that following IP injection of mice with LPS isolated from *Escherichia coli* 0111:B4, a potent cell shedding response is induced, similar to that observed in relapsing IBD patients [22]. In agreement with these studies, we found a significant increase in CC3-mediated cell shedding at 1.5 h post-injection of 1.25 mg kg^−1^ LPS, not only at the villus tip, but also along the shoulders and sides of the villus (figure 1*b*). Effects of LPS on the cell shedding response were found to be dose-dependent, in agreement with previous observations [22] (data not shown).

### 3.2. *Bifidobacterium breve* modulates lipopolysaccharide-induced cell shedding

**Figure 2.**
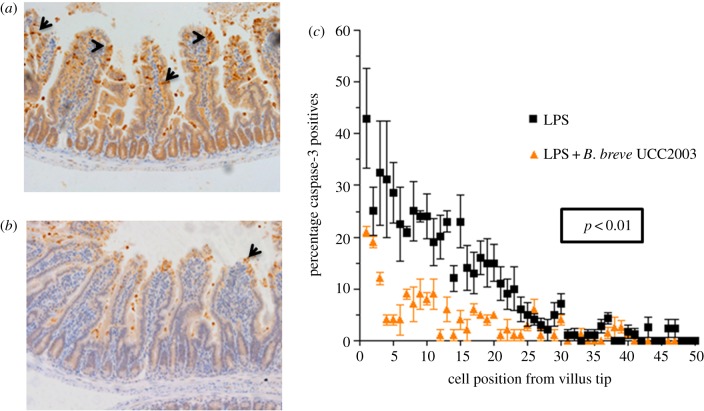
*Bifidobacterium breve* UCC2003 protects against LPS-induced cell shedding. C57 BL/6 mice received three daily oral gavage doses of (*a*) PBS or (*b*) approximately 1 × 10^9^ *B. breve* UCC2003 followed by IP challenge with LPS 24 h later. Representative images are shown. Formalin-fixed, paraffin-embedded intestinal sections were sectioned and stained with anti-CC3 and (*c*) quantified using the WinCrypts and Score programs, 20 well-orientated hemi-villi were counted per mouse. Data are mean ± s.d., *n* = 12 (two independent experiments) analysed with a Mann–Whitney *U*-test.

Various members of the microbiota are known to promote a healthy gut [29], although the precise mechanisms behind this remain incompletely understood. We reasoned that because the integrity of the intestinal epithelium is intrinsically linked to the well-being of the host and because the microbiota is expected to impact on epithelial cross-talk, such health-promoting species might play a role in regulating cell shedding. To test this, groups of C57 BL/6 mice were initially dosed with vehicle control (PBS) or with 1 × 10^9^ *B. breve* UCC2003 (isolated from a healthy infant) in 3 × 24 h doses orally to establish stable colonization [7]. Colonization was confirmed by faecal CFU counts on day 4 (electronic supplementary material, figure S1). Mice were then administered LPS to induce pathological cell shedding, followed by sacrifice at 1.5 h. Following dosing with *B. breve* UCC2003 and induction of cell shedding with LPS, mice showed a marked reduction in the levels of CC3-positive shedding cells compared with LPS-treated control mice receiving PBS gavage (figure 2*a,b*). Cell count analysis confirmed significant reduction (*p* < 0.01) in cell shedding at the majority of positions along the length of the villus in *B. breve* UCC2003-treated mice (figure 2*c*). Thus, *B*. *breve* appears to modulate epithelial integrity/survival during periods of inflammatory insult.

Previous studies have indicated that bifidobacteria may modulate the composition of other microbiota members, and within the context of IBD, studies have linked microbiota disturbances with active disease. Thus, to determine if bifidobacterial colonization impacts the gut microbiota, we analysed the community composition using a 16S rRNA-based sequencing approach. We found minor changes to the community structure in *B. breve* UCC2003 versus control treated mice (C57 BL/6), but overall, no notable differences (but expected increase in Actinobacteria in the *B. breve* UCC2003 group) in microbiota class abundance between the treatment groups (electronic supplementary material, figure S2). Bifidobacterial colonization takes place along the gastrointestinal (GI) tract including the small/large intestine and caecum. RNAscope analysis showed that *B. breve* UCC2003 was found in intimate contact with the IECs of the small intestine in colonized C57 BL/6 mice (electronic supplementary material, figure S3). Together, these data suggest that colonization with *B. breve* does not produce significant shifts in the overall gut microbiota community structure and that the observed protective effects after colonization are more likely to be related to direct effects of *B. breve*, possibly through interactions with the IECs.

### 3.3. The mechanism of protection against lipopolysaccharide-induced cell shedding is TNF-α independent

**Figure 3.**
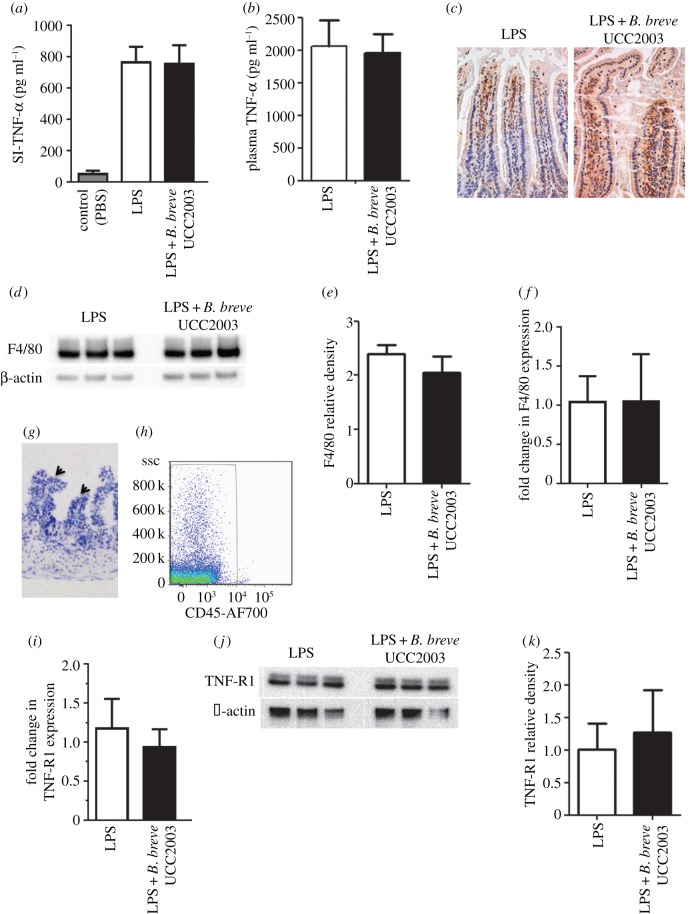
The cytoprotective effect of *B. breve* is not mediated by the TNF-α signalling pathway. C57 BL/6 mice were gavaged with PBS or *B. breve* and challenged with PBS or LPS for 1.5 h. Columns show TNF-α levels (via ELISA) in (*a*) whole small intestine intestinal homogenates or (*b*) plasma ± s.d. (*c*) Representative immunohistochemical staining for F4/80^+^ macrophages (brown cells) in control or *B. breve-*colonized mice. (*d*) Western blot analysis (F4/80 or housekeeping β-actin) of whole small intestinal homogenates, with (*e*) columns show relative density of F4/80 from (from *d*) whole intestinal homogenates. (*f*) Columns show F4/80 expression via RT-PCR ± SD. (*g*) Representative histology image of epithelial cell stripping protocol (modified Weiser method) leaving lamina propria intact (as indicated by arrows) and (*h*) FACS analysis for purity (anti-CD45). (*i*) Columns shown TNF-R1 expression via RT-PCR ± SD and (*j*) western blotting for protein expression in isolated intestinal epithelial cells, with (*k*) columns showing relative density of TNF-R1 (from (*j*)). *n* = 9 mice per group, representative of three experiments analysed with ANOVA Kruskal–Wallis test with Dunn's multiple comparison test (*a*), and with Mann–Whitney *U-*test (*b,e,f,i,k*).

LPS-induced cell shedding is caused by the release of TNF-α from lamina propria tissue-resident macrophages, which binds to TNF-receptor 1 (TNF-R1), on IECs [22], thereby driving the apoptotic response. Conditioning of macrophage responses by the microbiota has been reported previously [30] and, consistent with these data, bacteria such as *B. breve* have been described to possess immunomodulatory properties [31]. Thus, to determine whether the cell shedding outcome, as modulated by *B. breve*, was caused by reduced expression of TNF-α from macrophages, we isolated RNA and protein from whole small intestine of control and *B. breve* UCC2003-treated C57 BL/6 mice following LPS-mediated induction of cell shedding. As shown in figure 3*a*, no significant difference (*p* > 0.05) in levels of TNF-α protein was observed between groups, and this was confirmed at the transcriptional level (data not shown). We also found no significant changes (*p* > 0.05) in expression of TNF-α in the plasma of *B. breve* UCC2003-treated versus control mice following LPS-induced cell shedding (figure 3*b*), nor any significant difference (*p* > 0.05) in the numbers/levels of F4/80^+^ macrophages infiltrating the small intestine (figure 3*c–f*). Together, these data suggest that modulation of the reduced cell shedding response is independent of TNF-α induction. Because the microbiota may be able to interact directly with IECs, we postulated that *B. breve* modulates a signalling pathway downstream of the TNF-α ligand. To test whether expression of TNF-R1 was altered in the epithelium following dosing with *B. breve* UCC2003, IECs were isolated from whole small intestinal tissue using a modified Weiser methodology [32], after which purity of the IEC population was confirmed by histological analysis of stripped intestinal tissue and FACS analysis (figure 3*g,h*). Subsequent quantitative RT-PCR and western blot analysis of isolated IEC populations showed no significant changes (*p* > 0.05) to expression of the TNF-R1 transcript or protein following exposure to *B. breve* UCC2003 (figure 3*i–k*), suggesting that there is no impairment of signalling at the level of the receptor.

### 3.4. Functional epithelial MyD88 signalling is required for *Bifidobacterium breve*-mediated protection against cell shedding

IECs sample microbe-associated molecular patterns (MAMPS) of the intestinal luminal contents using a variety of receptors including members of the nucleotide-binding oligomerization domain (NOD) family, the C-type lectin receptor (CLR) family and the Toll-like receptor (TLR) superfamily. MyD88 is a critical adaptor protein in signalling downstream of the majority of the TLR family members [33]. We thus used epithelial-specific (Vil-Cre) MyD88 knockout mice to determine whether *B. breve* elicits its protective effects via epithelial TLR signalling pathways.

**Figure 4.**
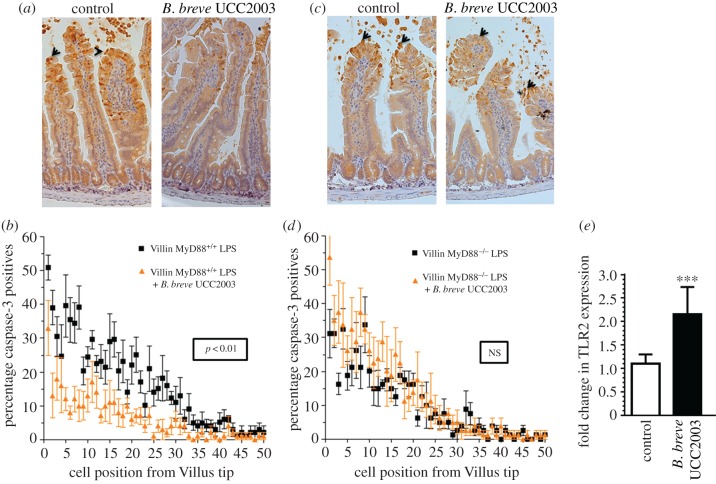
The cytoprotective effect of *B. breve* is MyD88 dependent. (*a,b*) IEC MyD88^+/+^ mice and (*c,d*) IEC MyD88^−/−^ mice were gavaged with PBS (control) or *B. breve* UCC2003 and challenged with LPS. Paraffin-embedded intestinal sections were stained with anti-CC3 and quantified using the WinCrypts and Score programs. (*e*) Columns shown TLR2 expression via RT-PCR. Data are mean ± s.d., *n* = 12 (two independent experiments) analysed with Mann–Whitney *U*-test.

C57 BL/6 MyD88^−/−^ villin-cre mice (i.e. IEC MyD88 KO mice) colonized with *B. breve* UCC2003, showed similar rates (*p* > 0.05) of LPS-induced cell shedding to PBS gavaged IEC MyD88^−/−^ mice. In comparison, control mice (i.e. C57 BL/6 MyD88^+/+^ villin-cre) showed the expected protection (*p* < 0.01) against cell shedding in the presence of *B. breve* UCC2003 (figure 4*a–d*). Furthermore, RT-PCR analysis of IEC homogenates showed increased expression (*p* < 0.001) of TLR2 in *B. breve* UCC2003-colonized mice when compared with control mice (i.e. PBS, figure 4*e*). Taken together, these data indicate that functional MyD88 signalling, potentially via TLR2 is required for modulating the protective effect of *B. breve* against cell shedding outcomes.

### 3.5. *Bifidobacterium breve* exopolysaccharide plays a role in modulating protection against lipopolysaccharide-induced cell shedding

**Figure 5.**
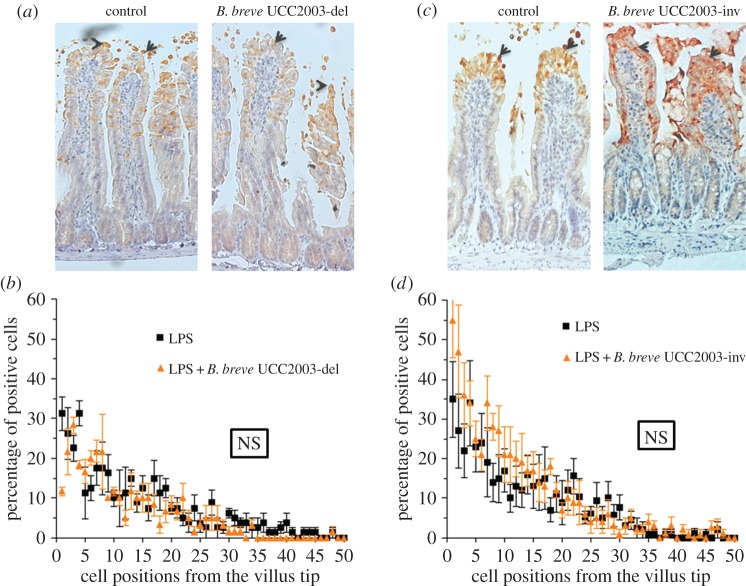
*Bifidobacterium breve* EPS plays a role in modulating the cytoprotective effect. C57 BL/6 mice were gavaged with either *B. breve* UCC2003 or (*a,b*) *B. breve* UCC2003del (i.e. EPS-negative) or (*c,d*) *B. breve* UCC2003inv (i.e. EPS2). Formalin-fixed, paraffin-embedded intestinal sections were stained with anti-CC3 and quantified using the WinCrypts and Score programs. Data are mean ± s.d., *n* = 12 (two independent experiments) analysed with Mann–Whitney *U*-test.

Recently, a number of functions modulated by bifidobacteria have been shown to be mediated through surface-associated EPS including resistance to gut infection [7]. Interestingly, the *eps* gene clusters represent a relatively conserved feature of bifidobacterial genomes, including those of the species *B. breve* [34]. In order to investigate the role of EPS in modulating the response against cell shedding, we used a deletion mutant (*B. breve* UCC2003-EPSdel) that expresses neither EPS1 nor EPS2 [7]. Mice were stably colonized by dosing with *B. breve* EPS-positive or EPS-negative strains followed by challenge with LPS (electronic supplementary material, figure S1). Strikingly, when colonized with the *B. breve* UCC2003-EPSdel, no significant protection (*p* > 0.05) against cell shedding was observed in control (i.e. PBS) versus colonized mice (figure 5*a,b*).

*Bifidobacterium breve* UCC2003 controls EPS biosynthesis via a bidirectional gene cluster which results in expression of either EPS1 (*B. breve* UCC2003) or EPS2 (*B. breve* UCC2003-EPSInv) [7]. Thus, to gain further insights into the role of a different EPS in the protective cell shedding response, we undertook studies using *B. breve* UCC2003-EPSInv. Colonization with EPS2 expressing *B. breve* (i.e. *B. breve* UCC2003-EPSInv) also failed to show any significant protection (*p* > 0.05) against LPS-induced cell shedding, suggesting considerable variation in the protective response dependent upon EPS genetic and chemical structure and organization (figure 5*c,d*). All strains are directly compared in electronic supplementary material, figure S4.

Together, these studies emphasize the striking strain variant specificity that is observed with regard to the individual protective effects of these bacteria following LPS-induced cell shedding. This is probably regulated by the specific molecules produced by each strain, including the EPS. This highlights the critical need to fully genetically characterize ‘probiotic’ strains of bacteria to enable a detailed dissection of their functional effects *in vivo* for optimal translation to human patients.

### 3.6. *Bifidobacterium breve* exopolysaccharide attenuates inflammatory and apoptosis signalling

**Figure 6.**
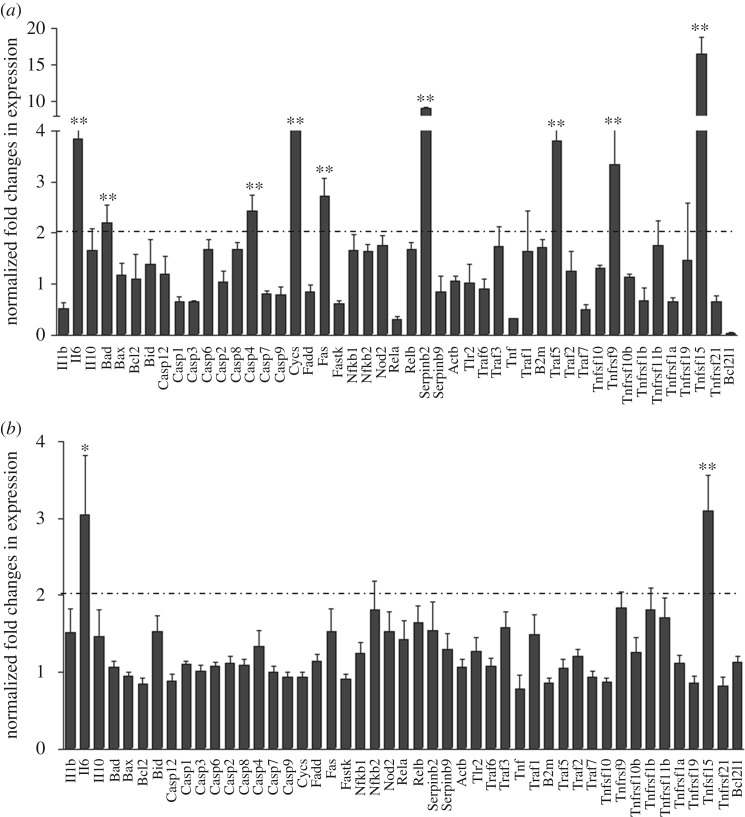
*Bifidobacterium breve* EPS attenuates inflammatory and apoptosis signalling. Whole small intestinal homogenates from LPS-challenged (*a*) *B. breve* UCC2003-EPSdel and (*b*) *B. breve*-colonized mice compared with control (i.e. PBS) were analysed using a custom RT-PCR array. Data are mean ± SD, *n* = 6 (two independent experiments), *p < 0.05 and **p < 0.01 and analysed with Mann–Whitney *U* test.

In order to gain further insights into the changes taking place in the small intestine following colonization with *B. breve* UCC2003 and the influence of EPS, whole small intestinal samples from control (i.e. PBS) and colonized (EPS-positive *B*. *breve* UCC2003 and EPS-negative *B*. *breve* UCC2003-del) mice following challenge with LPS were analysed using a custom RT-PCR array (figure 6: 49/84 targets are shown; full set of data is displayed in electronic supplementary material, figure S5) to look for transcriptional changes to key inflammatory transcripts and those involved in the apoptotic cascade. Interestingly, small intestinal samples from *B. breve* UCC2003-EPSdel-colonized mice (figure 6*a*; electronic supplementary material, figure S5*a,b*) showed significant increases (more than twofold and *p* < 0.01) in IL-6 and Tnfrs15 when compared with control and LPS-challenged mice. Moreover, numerous other apoptotic and inflammatory genes were significantly upregulated (more than twofold, *p* < 0.01) including Bad, Cycs (cytochrome *c*, Somatic), casp4, Fas, Traf5 and Tnfrs9. In contrast, in EPS-positive-colonized mice (i.e. *B. breve* UCC2003), our analysis showed only subtle changes to the expression of the majority of the targets when compared with PBS-treated control mice challenged with LPS (figure 6*b*; electronic supplementary material, figure S5c,*d*). In addition, while significant elevation (more than twofold and *p* < 0.05) in IL-6 and Tnfrs15 was observed following colonization with *B. breve* UCC2003*,* Tnfrs15 expression was markedly decreased versus *B. breve* UCC2003-EPSdel-colonized mice (threefold versus 16-fold increase). These data suggest that signalling via EPS may downregulate inflammatory and apoptotic networks, which would otherwise lead to elevated cell shedding.

## 4. Discussion

We report that colonization of mice with *B. breve* significantly reduces pathological/apoptotic epithelial cell shedding, through a previously unknown mechanism involving bifidobacterial EPS-MyD88 signalling.

The gut microbiota appears central to maintaining epithelial barrier integrity and, importantly, disturbances in the microbiota appear pivotal in IBD pathogenesis. Indeed, IBD patients (paediatric and adult cohorts) have been shown to possess a reduced overall microbiota diversity and reductions in specific genera including *Clostridium*, *Bacteroides, Faecalibacterium* and (of particular interest here) *Bifidobacterium* [6,15,35]. Previous clinical trials have shown that administration of bifidobacterial strains can reduce the incidence of relapse in patients suffering from IBD [36]. Following LPS-induced cell shedding, we observed that *a priori* administration of *B. breve* UCC2003 (which is a human-isolated strain, thus more translationally relevant) conferred a significant level of protection, which manifested as significantly reduced caspase-3 positivity within the villus epithelium (figure 2*a–c*). Previous studies have highlighted that bifidobacterial supplementation may also modulate the wider microbiota in mouse models [37]. However, our data indicate that while there are modest differences between PBS and *B. breve*-colonized mice (as indicated by taxa abundance), there are no notable differences (with high variability between animals), suggesting limited effects on overall microbiota profiles (electronic supplementary material, figure S2). These data therefore suggests a more direct link between bifidobacteria and maintenance of epithelial integrity in the prevention of intestinal inflammation.

Previous studies have indicated that *Bifidobacterium* predominantly colonizes the colon of infants and adults, as determined from faecal or mucosal scrapings, or biopsy samples [38,39]; however, in this work (using a murine model), we have described SI-specific responses. From a translational perspective, in humans, these protective cell shedding responses may result from bifidobacteria cross-talk in the lower SI. Although difficult to measure in humans, previous studies have indicated *Bifidobacterium* colonization in the lower SI (i.e. the ileum, as we observe in our model). Notably, select studies using ileostomy effluents and illeum biopsies have indicated bifidobacteria (specifically *B. animalis* subsp. *lactis* and *B. breve*, respectively) are present in this area of the infant and adult GI tract [40,41]. Therefore, in the human context, we may observe direct SI signalling via resident bifidobacteria and/or remote SI feedback signalling from colonic bifidobacteria epithelium cross-talk, which could be tested in future clinical intervention studies.

As previously mentioned, studies have shown that this experimental model of LPS-induced cell shedding is driven by an induction in expression of TNF-α from the intestinal mucosa [22,42]. One of the key functions of the gut microbiota is induction of tolerogenic or anti-inflammatory immune responses and thus we hypothesized that bifidobacteria may reduce cell shedding as a direct result of inhibiting TNF-α and macrophages—a potential source of TNF. However, we were unable to detect any changes in levels of TNF-α expression or macrophage infiltration from *B. breve* UCC2003-treated or control (i.e. PBS) mice (figure 3*a–f*), suggesting that the protection conferred by *Bifidobacterium* strains is TNF-α independent. Previous studies have indicated that colonization of *B. breve* UCC2003 during homeostatic conditions does not induce differences in splenic TNF-α-positive macrophage numbers when compared with non-colonized controls [7]. Coupled with the lack of change in expression in TNF-R1 following colonization (figure 3*i–k*), it appears that macrophages, TNF-α production and TNF-R1 signalling are not involved in modulating this protective response and suggests that *B. breve* UCC2003 acts preferentially from the luminal side through interactions with IECs. We cannot exclude the potential for EPS to block signalling via TNF-R1. However, TNF-R1 expression appears to be restricted to the basolateral surface of epithelial cells and thus *B. breve* would not be expected to have direct access to this cellular compartment for direct inhibition via binding [43]. Furthermore, quantification of downstream effectors (electronic supplementary material, figure S5) including FADD, TRAF2 and caspase 2 and 8 does not significantly differ between *B. breve* UCC2003 and *B. breve* UCC2003-del-colonized mice, which suggests EPS does not play a key role via TNF-R1.

To delineate these protective luminal bifidobacterial–epithelial interactions, we used epithelial-specific MyD88 KO mice; MyD88 is a key adaptor protein downstream of microbe-TLR signalling. Notably, mice carrying truncated epithelial MyD88 (i.e. C57 BL/6 MyD88^−/−^ villin-cre) showed no protection against cell shedding after colonization of *B. breve* UCC2003 (figure 4*c,d*); this was in stark contrast to MyD88-positive control animals, which again showed significant protection against LPS-induced cell shedding (figure 4*a,b*). Furthermore, we observed significant increases in IEC TLR2 expression in *B. breve* UCC2003-colonized mice (figure 4*e*). Interestingly, previous work has indicated that TLR2 may enhance ZO-1 associated intestinal epithelial barrier integrity [44], and other studies indicate that mice deficient in MyD88 signalling have increased susceptibility to intestinal inflammation [12]. In a UV model of apoptosis, MyD88 signalling appears to reduce caspase-3 and in turn increase cell survival, and more recently *B. bifidum* has been shown to reduce apoptosis *in vitro* (necrotizing enterocolitis IEC-6 cell model), as also indicated by reduced CC3-positive cells [45]. Thus, our data, in tandem with these studies, indicate that *B. breve* UCC2003 may regulate epithelial integrity in response to LPS-induced cell shedding (as marked by caspase-3) via these central MyD88 signalling mechanisms, potentially downstream of TLR2.

Having determined the importance of host adaptor MyD88, we next sought to determine if there was a specific bifidobacterial molecule central to the observed protective response. Because we have previously shown that surface EPS of *B. breve* UCC2003 can regulate the host response [7], we investigated the ability of an EPS mutant *B*. *breve* UCC2003-EPSdel (complete deletion of *eps* biosynthetic cluster) to modulate LPS-induced cell shedding. Notably, mice receiving *B. breve* UCC2003-EPSdel showed no significant protection against cell shedding when compared with EPS-positive (i.e. *B. breve* UCC2003) colonized mice (figure 5*a,b*), suggesting an important role for this EPS in microbe–host cross-talk. Importantly, EPS structures can be recognized via TLR2 (and signal via MyD88), and previous work with the polysaccharide A (PSA) capsule of *Bacteroides fragilis* highlights that PSA can modulate dendritic cell and T regulatory cell function via TLR2 signalling [46,47]. Additionally, previous work has highlighted that a strain of *B. breve* (Yakult strain) can also induce IL-10 producing T regulatory cells via TLR2; however they did not determine if this was via an EPS-specific mechanisms [48]. Furthermore, recent studies using *Bacillus subtilis* have demonstrated that the EPS capsule of this bacterium is able to protect against intestinal inflammation in a murine model of colitis (in this instance via TLR4), providing further support for the likely role of bifidobacterial EPS in the effects observed in these studies [49]. Notably, the probiotic genus *Lactobacillus* also produces distinct EPSs, which are structurally similar to those observed in bifidobacteria [50]*.* Recently, within an *in vitro* system (HT29-19A epithelial cell line), the EPS from *Lactobacillus acidophilus* 5e2 was shown to increase IL-8 expression and also TLR2 expression (we also observe that *B. breve* UCC2003 induces IEC TLR2 expression), and additionally upregulation of TLR2 was found to potentially ‘sensitize’ epithelial cells to subsequent stimulation with peptidoglycan (a TLR2 agonist) [51]. Furthermore, the authors also observed a modest increase in TLR4 expression after addition of EPS, but did not detect any significant modulation of IL-8 responses after priming with EPS and subsequent addition of LPS, which may indicate less of a role for EPS–TLR4 interactions [51]. From a more systemic perspective, in the instance that *Lactobacillus* or indeed *B. breve* UCC2003 potentially translocate across the epithelial barrier, it could by hypothesized they directly influence macrophage function. Previous studies have shown that *L. casei* Shirota can dampen down inflammatory macrophage responses, and *L. rhamnosus* EPS has also been shown to modulate macrophage function *in vitro*, but on this occasion induced proinflammatory responses [52,53]. Ideally, we would test our *B. breve* strains in TLR2 and/or TLR4 KO animals; unfortunately, previous work has shown that these mice do not respond to LPS and thus would not have a cell shedding response, making these further studies not possible. However, in studies using RNAscope, we found significant numbers of *B. breve* UCC2003 associated with the villi in colonized mice (electronic supplementary material, figure S3), suggesting that direct signalling interactions between the bacteria (possibly via EPS and TLRs, and *B. breve* UCC2003 colonization increases TLR2 expression) and IECs may play an important role in modulating this response. These data, alongside our findings, suggest that *B. breve* EPS may regulate cell shedding by acting as TLR ligands via MyD88, leading to protective epithelial responses.

To probe these EPS–epithelial interactions further, we took advantage of the bidirectional *eps* gene cluster in *B. breve* UCC2003, which can express two genetically and importantly chemically distinct surfaces EPSs [7]. All previous studies used EPS1 (i.e. with *B. breve* UCC2003), but we also determined responses following EPS2 (i.e. *B. breve* UCC2003-EPSInv) colonization. Strikingly, and contrary to our expectations, we found that this isogenic strain was unable to confer protection against LPS-induced cell shedding (figure 5*c,d*). Importantly, EPSs are composed of repeating mono- or oligosaccharides linked by various glycosidic linkages, and the three-dimensional structures and other physiochemical features of EPSs can vary widely [54]. The variability in chemical composition of these two *B. breve* EPSs (previous work suggests the EPSs may include glucose, galactose and/or the *N*-acetylated versions of these two sugars in different ratios or composition [7]) could, in part, explain the different modulatory properties of this molecule in relation to receptor-ligand binding, and further highlights the issues with significant strain (or in this case isogenic), variation in effects on host responses. Importantly, these different EPS-epithelium protective responses do not appear to be linked to colonization ability as all strains colonized mice at similar levels (electronic supplementary material, figure S1). Previous limited studies have indicated that specific chemical structures of EPSs such as PSA of *B. fragilis* (comprised an unusual repeating tetrasaccharide moiety, free carboxyl, phosphate and amino groups, that contribute to its zwitterionic nature) are important for function [46]. Additionally, *in vitro* studies on *L. reuteri* strains (DSM 17938 and L26 Biocenol) indicate both EPSs are high-molecular-weight d-glucan polysaccharides with differing spatial conformations, which may relate to induction of different cytokine responses. However, the direct chemical structures involved in this modulation have yet to be defined [55]. Future challenges will include studies to fully chemically characterize the different strains of ‘probiotic’ bacteria, as evidently significant differences in response to small strain variations (including variations in EPS expression and structure and also other MAMPS) may impact beneficial host responses [56,57].

We have previously shown that EPS-positive *B. breve* UCC2003 does not induce inflammatory host responses after colonization, which we hypothesize is to the advantage of the bacterium and host for maintaining efficient symbiosis and homeostasis [7]. Interestingly, when we probed the downstream signalling transcriptional events after colonization and LPS challenge, we determined that presence of EPS1 (i.e. *B. breve* UCC2003) appeared to attenuate apoptosis-induced signalling activation, in stark contrast to mice colonized with the *B. breve* UCC2003-EPSdel strain, which had significantly elevated apoptotic gene expression (figure 6; electronic supplementary material, figure S5). Importantly, previous work has demonstrated that activation of MyD88 can downregulate several of these genes including Fas (CD95) [58]. Fas is a cell surface receptor and member of the TNF superfamily, and when bound by its ligand induces apoptosis through the assembly of a multiprotein complex called DISC, which in turn activates caspase 8 (i.e. extrinsic apoptosis pathway) [59]. Further evidence of an EPS-specific mechanism attenuating epithelial apoptosis comes from the observation that Bad, Cycs, casp4, Traf5 and Tnfr9 are upregulated in the intestinal mucosa of mice colonized by *B. breve* UCC2003-del compared with *B. breve* UCC2003-colonized mice. Bad is a pro-apoptotic (BH3-only) member of the bcl-2 family that antagonizes the anti-apoptosis proteins bcl-2, bcl-xl and bcl-2, allowing activation of bax/bak oligomers and release of cytochrome *c* from the mitochondria. Within the same pathway, Cycs encodes the haem protein cytochrome *c*, which forms a multiprotein complex called the apoptosome, which activates a cascade of caspases which cause apoptotic cell death [60]. Traf5 is a scaffold protein that forms a multiprotein complex with TRAF2, RIP1 and the TNF receptor, and can potentially mediate the activation of apoptosis and NF-κB [61]. We have previously shown that NF-κB1 inhibits LPS-induced apoptotic cell shedding, whereas NF-κB2 stimulates apoptotic cell shedding [22]. TNFRF9 (CD137) is expressed on T cells and has been reported to enhance their cytolytic activity [62]. These data strongly suggest that, mechanistically, *B. breve* UCC2003, via EPS, may block intrinsic and extrinsic apoptosis signalling (via activation of MyD88) during inflammation to protect epithelial cells under highly apoptotic conditions.

In summary, we have demonstrated that certain bifidobacteria (i.e. *B. breve* UCC2003) are able to protect against pathologic cell shedding induced by IP injection of LPS, and that this protection appears to be independent of TNF-α production by resident tissue macrophages. Using wild-type and mutant *B. breve*, we have demonstrated that a specific EPS is able to confer this protection and, using knockout mice, have shown that this protection appears contingent on functional (MyD88) signalling downstream of the epithelial TLR family members and modulation of pro-apoptotic gene pathways. Understanding how health-promoting species of bacteria such as the *Bifidobacterium* genus interact with the intestinal epithelium and how these species confer their protective effects may drive progress towards understanding how pathologic cell shedding in IBD patients is linked to changes in the intestinal microbiota and how intervention strategies could positively impact disease progression. Future human studies could be considered to address issues of microbial dysbiosis, the relationship to the cell shedding response, to what extent microbial dysbiosis is linked to periods of remission and relapse in such patients, and how bifidobacterial supplementation could be used to reduce relapse in IBD patients.

## Supplementary Material

Supplementary Methods & Figures

## Data accessibility

The datasets supporting this article have been uploaded as part of the electronic supplementary material.
