# Supplementary material for: Bifidobacterium breve reduces apoptotic epithelial cell shedding in an exopolysaccharide and MyD88-dependent manner
Source: Open Biol. 2017 Jan 25;7(1):160155. doi: 10.1098/rsob.160155 (PMC5303268; doi:10.1098/rsob.160155)
Supplement: Supplementary Methods & Figures [file rsob160155supp1.pdf]

Supplementary Methods & Figures for manuscript:

"*Bifidobacterium breve* reduces apoptotic epithelial cell shedding in an exopolysaccharide and MyD88-dependent manner"

K.R. Hughes, L.C. Harnisch, C. Alcon-Giner, S. Mitra, C.J. Wright, J. Ketskemety, D. van Sinderen, A.J.M Watson and L.J. Hall

Supplementary Materials and Methods

RNAscope

RNAscope was performed using a commercial kit (RNAscope FFPE reagent kit) from Advanced Cell Diagnostics (California, USA) as per the manufacturer's instructions. Briefly, 5 µm formalin fixed paraffin embedded small intestinal tissue was mounted on Superfrost plus slides (ThermoFisher) before baking in a dry oven at 60°C for 1 h. Slides were then deparaffinised with Xylene and 100% ethanol before applying Pre-treat solution 1 for 10 minutes at room temperature. Slides were then washed in distilled water before incubating in boiling Pre-treat 2 solution for 15 minutes. Following further washes, Pre-treat solution 3 was applied in a humidified chamber at 40°C for 30 minutes. After further washes, *B. breve* UCC2003 specific probe (probe name: B-Bifido-16SrRNA) or Cyclophylin B control probe was hybridised to the slides for 2 h at 40°C. Following washing in wash buffer, a series of amplification probes (AMP1 to AMP6) were sequentially bound and washed to/from the slides before signal detection using DAB substrate as per the manufacturer's recommendations. Slides were then counterstained with haematoxylin, dehydrated and mounted for visualisation.

Faecal DNA extractions, quantification and sequencing

DNA was extracted from murine faecal samples using the FastDNA™ SPIN Kit for Soil (MP Biomedicals) following the manufacturer's instructions but incorporating an extension of the initial bead-beading time to three minutes. The concentration of bacterial DNA was quantified using Qubit and normalised to 5 ng ml^{-1} for all samples. Extracted DNA was used as a template for PCR amplification of the V4 region of the 16S rRNA gene, 5' AAT GAT ACG GCG ACC ACC GAG ATC TAC A and, 5' CAA GCA GAA GAC GGC ATA CGA GAT AAC T. Amplification conditions of the PCR were: 1 cycle of 94°C 3 min and 25 cycles of 94°C for 45 s, 55°C for 15 s and 72°C for 30 s using a 96 well Thermal Cycler PCR machine. 16S RNA gene libraries were sequenced on the Illumina MiSeq platform with 250 bp paired end reads.

Sequence processing

All raw sequence reads were processed through quality control using FASTX-Toolkit [1] keeping a minimum quality threshold of 33 for at least 50% of the bases. Reads that passed the threshold were aligned against SILVA database (version: SILVA_119_SSURef_tax_silva) [2] using BLASTN (ncbi-blast-2.2.25+; Max e-value 10e-3) [3] separately for both pairs. After performing the BLASTN alignment, all output files were imported and annotated using the paired-end protocol of MEGAN [4].

Taxonomic annotation

For processing the BLAST files by MEGAN6, we used parameter settings of "Min Score = 50", "Top Percent = 10". Some reads which did not have any match to the respective database were placed under a "No hit" node, and some reads that were originally assigned to a taxon that did not meet our selected threshold criterion were pushed back using the lowest common ancestor (LCA) algorithm to higher nodes where the threshold was met. After importing datasets in MEGAN, we obtained MEGAN-own "rma files" for each data mapped onto NCBI taxonomy based on our selected threshold. Further, all the files were compared and analysed within MEGAN.

Statistical analysis

For microbiota analysis R software was used [5]. Average community profile comparison of two groups are displayed using pie charts in MEGAN. Abundance matrices were depicted using boxplots in R for each taxa showing comparison of two groups.

References

1 Hannon, L. FASTX-Toolkit. 2010 [cited; Available from: http://hannonlab.cshl.edu/fastx_toolkit/

2 Quast, C., Pruesse, E., Yilmaz, P., Gerken, J., Schweer, T., Yarza, P., Peplies, J., Glockner, F. O. 2013 The SILVA ribosomal RNA gene database project: improved data processing and web-based tools. *Nucleic Acids Res*. **41**, D590-596. (10.1093/nar/gks1219)

3 Altschul, S. F., Gish, W., Miller, W., Myers, E. W., Lipman, D. J. 1990 Basic local alignment search tool. *J Mol Biol*. **215**, 403-410. (10.1016/S0022-2836(05)80360-2)

4 Huson, D. H., Mitra, S., Ruscheweyh, H. J., Weber, N., Schuster, S. C. 2011 Integrative analysis of environmental sequences using MEGAN4. *Genome Res*. **21**, 1552-1560. (10.1101/gr.120618.111)

5 Team, R. D. C. 2008 *R: A language and environment for statistical computing. R Foundation for Statistical Computing*. Vienna, Austria

Supplementary Figures

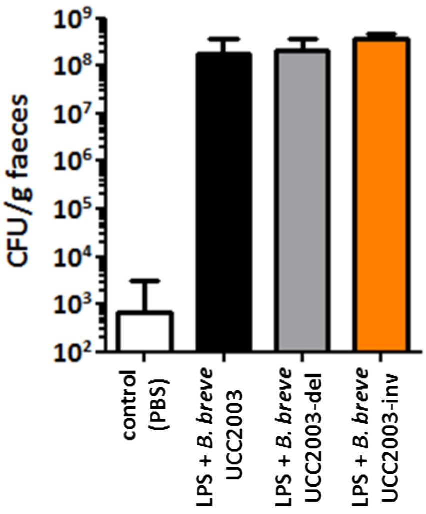

Supplementary Figure 1: *B. breve* strains stably colonise murine GI tract.

Faecal samples were collected from control (i.e. PBS), *B. breve* UCC2003, *B. breve* UCC2003-del and *B. breve* UCC2003-inv colonised mice on day 4 (after 3 x 24 h doses at ~1 x 10^9) and plated on RCA (+ mupirocin) and CFU enumerated at 24 hours.

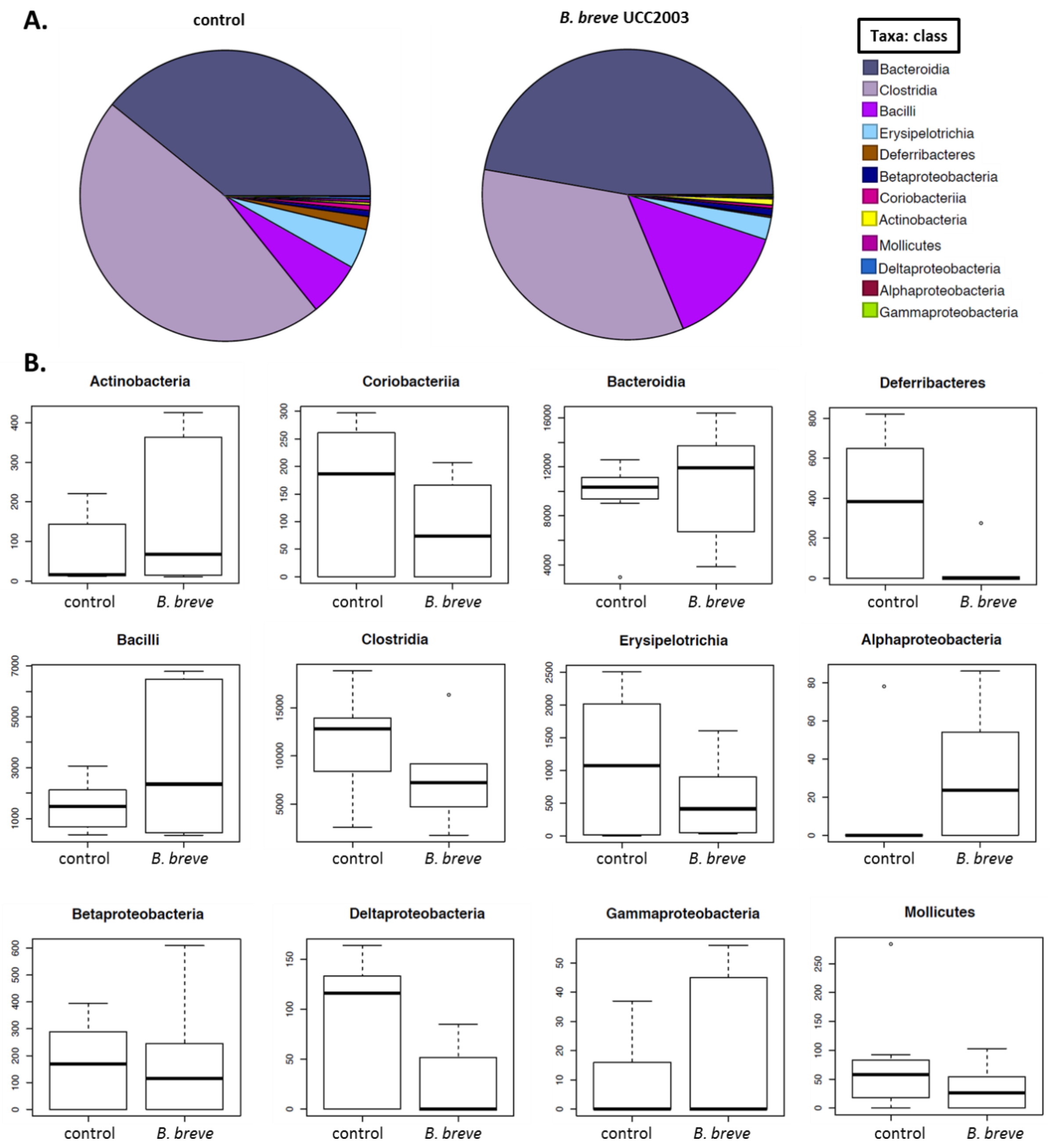

<u>Supplementary Figure 2: *B. breve* UCC2003 does not notably impact faecal microbiota profiles.</u> Faecal samples from control (i.e. PBS) and *B. breve* UCC203 (after 3 x 24 h doses at ~1 x 10^9) were collected and processed for 16s Illumina sequencing. **(A)** Pie chart depicting comparison of average taxonomic content for two groups at class level taxonomic profile. **(B)** Individual boxplot of each taxa at class level taxonomic profile for two groups (i.e. control and *B. breve* UCC2003), where read abundance data was normalised for both the groups.

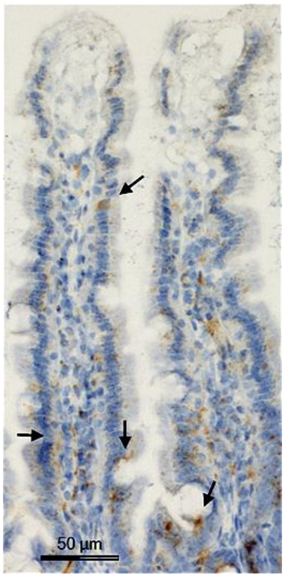

Supplementary Figure 3: *B. breve* UCC2003 resides in close contact with the small intestinal epithelium.

Representative RNAscope staining for *B. breve* (*B. breve* specific probe) brown cells and arrows) in the small intestine of *B. breve* colonised mice (after 3 x 24 h doses at ~1 x 10^9).

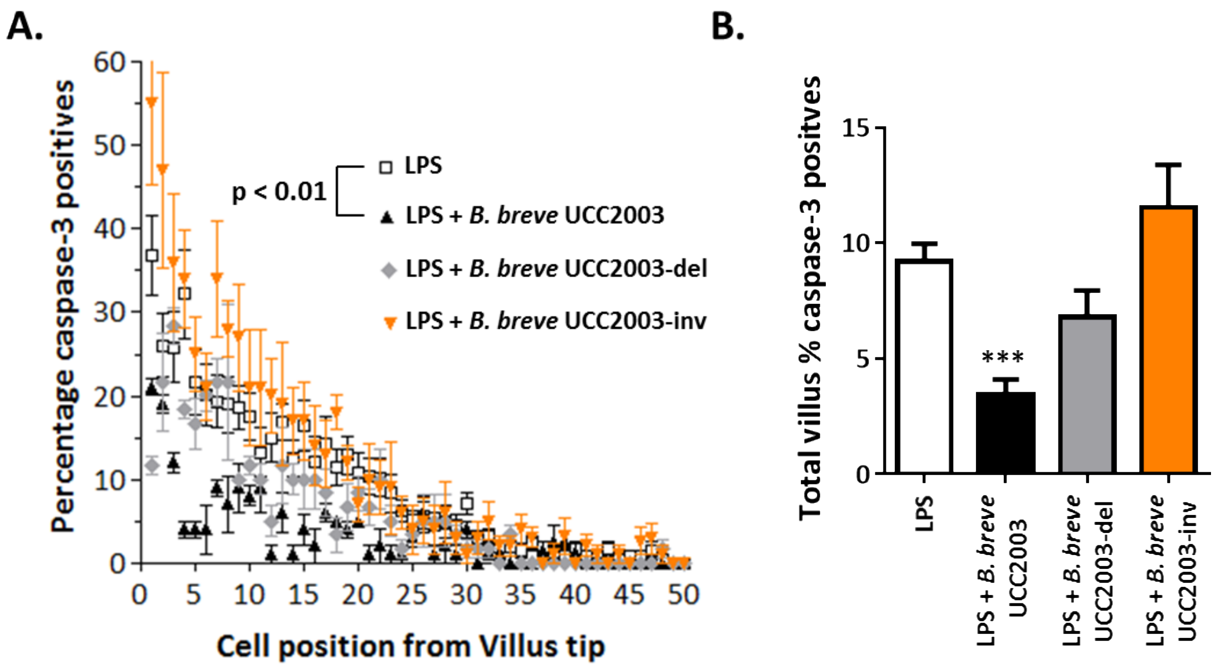

Supplementary Figure 4: Bifidobacterial EPS modulates cell shedding.

Combination of counts from Figures 2, 4 and 5 to provide representative overview of LPS-induced cell shedding responses between *B. breve* strains. C57 BL/6 mice were gavaged (3 x 24 h doses at ~1 x 10^9) with either *B. breve* UCC2003 or *B. breve* UCC2003del (i.e. EPS-negative) or *B. breve* UCC2003inv (i.e. EPS2) and challenged with LPS. **(A)** Formalin fixed, paraffin-embedded intestinal sections were stained with anti-CC3 and quantified using the WinCrypts and Score programs along villus length, **(B)** average percentage of total caspase-3 positive events. Data are mean ± SD, *n* = 12/group (two independent experiments) analysed with Mann–Whitney U test.

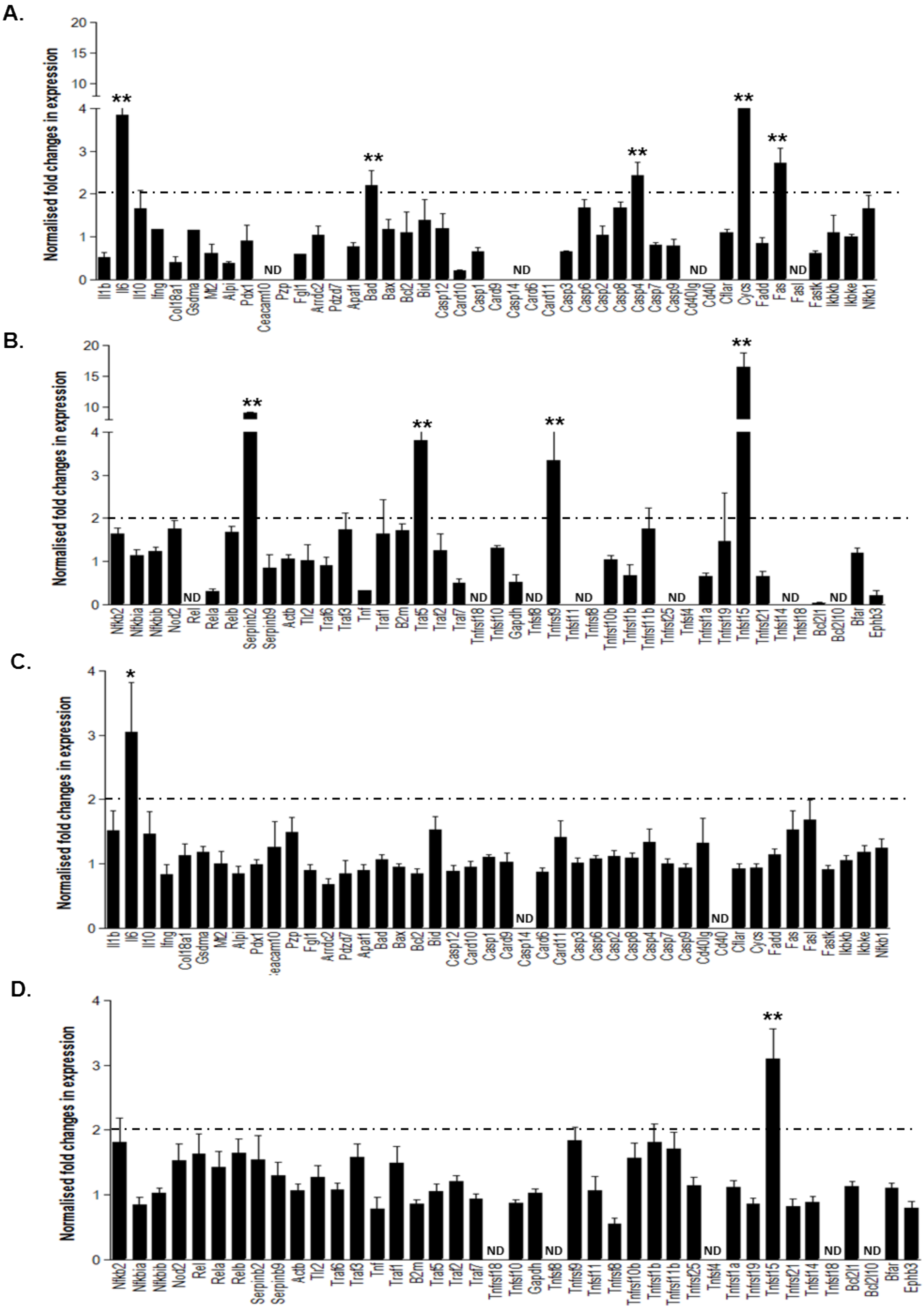

Supplementary Figure 5: *B. breve* EPS plays a role in modulating the cytoprotective effect.

Whole small intestinal homogenates from LPS challenged **(A, B)** *B. breve* UCC2003-EPSdel and **(C, D)** *B. breve* colonised mice compared to control (i.e. PBS) were subjected to custom array RT-PCR. Data are mean ± SD, $n = 6$ (two independent experiments), *$P < 0.05$ and **$P < 0.01$, non-detectable (ND), and analysed with Mann–Whitney U test
